# The mRNA decapping machinery targets *LBD3/ASL9* to mediate apical hook and lateral root development

**DOI:** 10.26508/lsa.202302090

**Published:** 2023-06-29

**Authors:** Zhangli Zuo, Milena E Roux, Jonathan R Chevalier, Yasin F Dagdas, Takafumi Yamashino, Søren D Højgaard, Emilie Knight, Lars Østergaard, Eleazar Rodriguez, Morten Petersen

**Affiliations:** 1 Department of Biology, Faculty of Science, University of Copenhagen , Copenhagen, Denmark; 2 Gregor Mendel Institute, Austrian Academy of Sciences, Vienna BioCenter, Vienna, Austria; 3 Laboratory of Molecular Microbiology, School of Agriculture, Nagoya University, Nagoya, Japan; 4 Crop Genetics Department, John Innes Centre, Norwich Research Park, Norwich, UK

## Abstract

Multicellular organisms perceive and transduce multiple cues to optimize development. Key transcription factors drive developmental changes, but RNA processing also contributes to tissue development. Here, we report that multiple decapping deficient mutants share developmental defects in apical hook, primary and lateral root growth. More specifically, *LATERAL ORGAN BOUNDARIES DOMAIN 3 (LBD3)/ASYMMETRIC LEAVES 2-LIKE 9 (ASL9)* transcripts accumulate in decapping deficient plants and can be found in complexes with decapping components. Accumulation of *ASL9* inhibits apical hook and lateral root formation. Interestingly, exogenous auxin application restores lateral roots formation in both *ASL9* over-expressors and mRNA decay–deficient mutants. Likewise, mutations in the cytokinin transcription factors type-B ARABIDOPSIS RESPONSE REGULATORS (B-ARRs) *ARR10* and *ARR12* restore the developmental defects caused by over-accumulation of capped *ASL9* transcript upon *ASL9* overexpression. Most importantly, loss-of-function of *asl9* partially restores apical hook and lateral root formation in both *dcp5-1* and *pat* triple decapping deficient mutants. Thus, the mRNA decay machinery directly targets *ASL9* transcripts for decay, possibly to interfere with cytokinin/auxin responses, during development.

## Abstract (precis)

This study identifies *LBD3/ASL9* as targets of mRNA decay machinery, and *ASL9* contributes to apical hook and lateral root development, possibly through interfering with cytokinin/auxin responses.

## Introduction

Understanding proper tissue development requires information about diverse cellular mechanisms controlling gene expression. Much work has focused on the transcriptional networks that govern stem cell differentiation. For example, ectopic expression of *LATERAL ORGAN BOUNDARIES DOMAIN (LBD)/ASYMMETRIC LEAVES 2-LIKE (ASL)* genes is sufficient to induce spontaneous proliferation of pluripotent cell masses in plants, a reprogramming process triggered in vitro by complementary/Yin-Yang phytohormones auxin and cytokinin (Fan et al, 2012; Schaller et al, 2015). Auxin and cytokinin responses are essential for a vast number of developmental processes in plants including postembryonic reprograming and formation of the apical hook to protect the meristem during germination in darkness (Chaudhury et al, 1993; Hu et al, 2017) and lateral root (LR) formation (Jing & Strader, 2019). Loss-of-function mutants in genes that regulate auxin-dependent transcription such as *auxin-resistant1 (axr1)* exhibit defective hooking and LR formation (Estelle & Somerville, 1987; Lehman et al, 1996). In addition, type-B ARABIDOPSIS RESPONSE REGULATORS (B-ARRs) ARR1, ARR10, and ARR12 work redundantly as transcriptional activators to regulate cytokinin targets including type-A ARRs, which are negative regulators of cytokinin signaling in shoot development and LR formation (Riefler et al, 2006; Ishida et al, 2008; Xie et al, 2018). Exogenous cytokinin application disrupts LR initiation by blocking pericycle founder cell transition from G2 to M phase (Li et al, 2006; Laplaze et al, 2007). Thus, reshaping the levels of certain transcription factors leads to changes in cellular identity. As developmental programming must be tightly regulated to prevent spurious development, the expression of these transcription factors may be controlled at multiple levels (Tatapudy et al, 2017). However, most developmental studies focus on their transcription rates and overlook the contribution of mRNA stability or decay to these events (Crisp et al, 2016).

Eukaryotic mRNAs contain stability determinants including the 5′ 7-methylguanosine triphosphate cap (m7G) and the 3′ poly-(A) tail. mRNA decay is initiated by deadenylation, followed by degradation via either 3′–5′ exosomal exonucleases and SUPPRESSOR OF VCS (SOV)/DIS3L2 or via the 5′–3′ exoribonuclease (XRN) activity of the decapping complex (Garneau et al, 2007; Sorenson et al, 2018). This complex includes the decapping holoenzyme composed of the catalytic subunit Decapping 2 (DCP2) and its cofactor DCP1 along with other factors (DCP5, DHH1, VCS, LSM1-7 complex, and PAT1), and the XRN that degrades monophosphorylated mRNA. As a central platform, PAT1 (Protein Associated with Topoisomerase II, PAT1b in mammals) forms a heterooctameric complex with LSM (Like-sm)1–7 at 3′ end of a mRNA to engage transcripts containing deadenylated tails thereafter recruits other decapping factors and interacts with them using different regions; these decapping complex and mRNAs can aggregate into distinct cytoplasmic foci called processing bodies (PBs) (Brengues et al, 2005; Balagopal & Parker, 2009; Ozgur et al, 2010; Chowdhury et al, 2014; Charenton et al, 2017; Lobel et al, 2019). Beyond *DCP* genes, deletion of *PAT1* gene in yeast exhibits the strongest temperature sensitive phenotype compared with other decapping factors genes (Bonnerot et al, 2000).

mRNA decay regulates mRNA levels and thereby impacts cellular reprogramming (Newman et al, 2017; Essig et al, 2018). We and others have shown that the decapping machinery is involved in stress and immune responses (Xu & Chua, 2012; Merret et al, 2013; Roux et al, 2015; Perea-Resa et al, 2016; Crisp et al, 2017; Yu et al, 2019), and that RNA-binding proteins can target selected mRNAs for decay (Gerstberger et al, 2014; Perea-Resa et al, 2016; Yu et al, 2019). Postembryonic lethality (Xu et al, 2006) and stunted growth phenotypes (Xu & Chua, 2009; Perea-Resa et al, 2012) associated with disturbance of the decay machinery indicate the importance of mRNA decapping and decay machinery during plant development. However, although much has been learned about how mRNA decapping regulates plant stress responses (Perea-Resa et al, 2016; Yu et al, 2019; Zuo et al, 2021), far less is known about how decapping contributes to plant development.

*Arabidopsis dcp1*, *dcp2*, and *vcs* mutants display postembryonic lethality, whereas *lsm1alsm1b*, *pat* triple mutant, and *dcp5* knockdown mutants only exhibit abnormal development (Xu et al, 2006; Xu & Chua, 2009; Perea-Resa et al, 2012; Zuo et al, 2022a, 2022b *Preprint*). All these differences suggest that mutations in mRNA decay components may cause pleiotropic phenotypes not directly linked to mRNA decapping and decay deficiencies (Riehs-Kearnan et al, 2012; Gloggnitzer et al, 2014; Roux et al, 2015). For example, it has been proposed that lethality in some mRNA decay loss-of-function mutants is not due to decay deficiencies per se but to the activation of immune receptors which evolved to surveil microbial manipulation of the decay machinery (Roux et al, 2015). In line with this, loss-of-function of *AtPAT1* inappropriately triggers the immune receptor SUMM2, and *Atpat1* mutants consequently exhibit dwarfism and autoimmunity (Petersen et al, 2000; Zhang et al, 2012; Roux et al, 2015; Rodriguez et al, 2020). Thus, PAT1 is under immune surveillance and PAT proteins are best studied in SUMM2 loss-of-function backgrounds.

Here, we studied the impact of mRNA decapping during development. For this, we have analyzed three sequential mRNA decapping mutants *dcp2-1*, *dcp5-1*, and *pat* triple mutant (*pat1-1path1-4path2-1summ2-8*), revealing that the mRNA decay machinery targets the important developmental regulator *ASL9*. Specifically, disruption of the mRNA decay machinery promotes *ASL9* accumulation, and this in turn contributes to inhibit apical hook and lateral root formation. Interestingly, these developmental defects, which are observed in mRNA decapping deficient mutants and *ASL9* over-expressors, can be salvaged through disruption of cytokinin signaling or exogenous application of auxin. Importantly, mutations in *asl9* also partially restores the developmental defects including apical hook and lateral root formation in decapping mutants. These observations indicate that the mRNA decay machinery is fundamental to developmental decision-making.

## Results

### mRNA decapping deficiency causes deregulation of apical hooking

**Figure 1.**
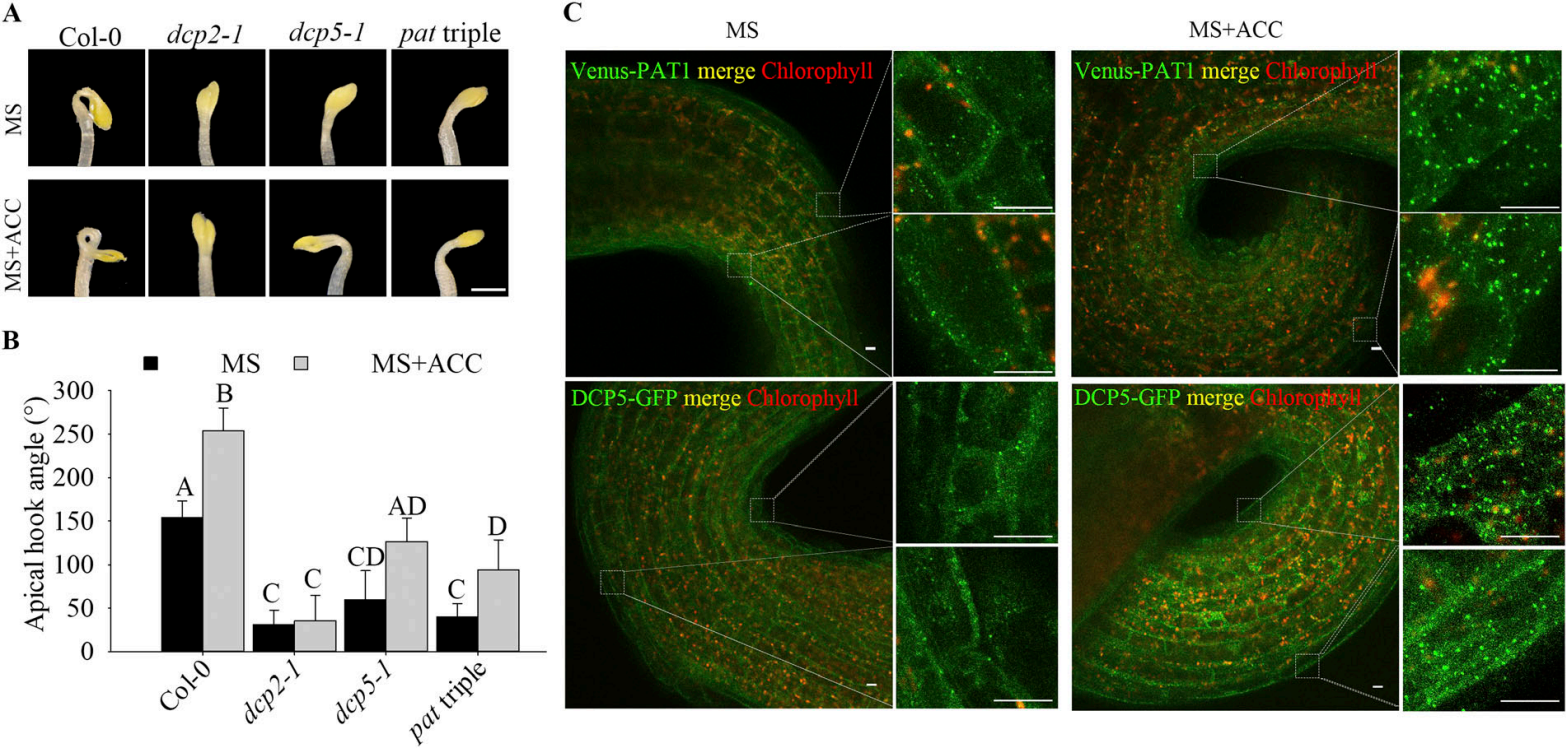
mRNA decapping deficiency causes deregulation of apical hooking. **(A, B)** Hook phenotypes (A) and apical hook angles (B) in triple response to ACC treatment of etiolated Col-0, *dcp2-1*, *dcp5-1*, and *pat* triple seedlings. The experiment was repeated three times, in each repeat sample size (n)>30 for each genotype and treatment, and representative pictures are shown. The scale bar indicates 1 mm. Bars marked with the same letter are not significantly different from each other (*P*-value > 0.05). **(C)** Representative confocal microscopy pictures of hook regions following ACC treatment. Dark-grown seedlings with either Venus–PAT1 (top) or DCP5–GFP (bottom) on MS or MS + ACC plates for 4 d. Scale bars indicate 10 μm.

**Figure S1.**
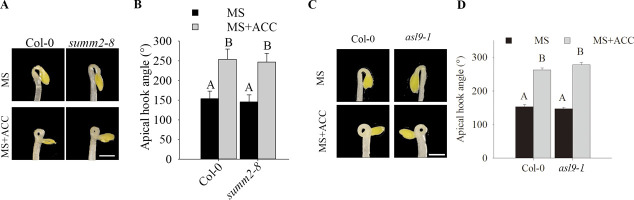
Apical hook in *summ2-8* and *asl9-1* mutants. **(A, B)** Hook phenotypes (A) and apical hook angles (B) in triple responses to ACC treatment of etiolated seedlings of Col-0 and *summ2-8*. **(C, D)** Hook phenotypes (C) and apical hook angles (D) in triple responses to ACC treatment of etiolated Col-0 and *asl9-1* seedlings. The treatment was repeated three times (n > 30), and representative pictures are shown. The scale bar indicates 1 mm. Bars marked with the same letter are not significantly different from each other (*P*-value > 0.05).

We and others have reported that mutants of mRNA decay components exhibit abnormal developmental phenotypes including postembryonic death and stunted growth (Xu et al, 2006; Xu & Chua, 2009; Perea-Resa et al, 2012; Roux et al, 2015; Zuo et al, 2022b *Preprint*), indicating mRNA decay may be needed for proper development. To assess this, we explored readily scorable phenotypic evidence of defective development. Because apical hooking can be exaggeratedly induced by exogenous application of ethylene or its precursor ACC, we germinated seedlings in darkness in the presence or absence of ACC (Bleecker et al, 1988; Guzman & Ecker, 1990). Interestingly, all three sequential mRNA decapping mutants tested *dcp2-1*, *dcp5-1*, and *pat* triple mutant were hookless and unable to make the exaggerated apical hook under ACC treatment (Figs 1A and B and S1A and B), being that *dcp2-1* exhibit the strongest hookless phenotype. Because *dcp2-1* is postembryonic lethal, we used seeds from a parental heterozygote to score for hook formation, and subsequentially confirmed by genotyping that all hookless seedlings were *dcp2-1* homozygotes. This, and the fact that ACC treatment leads to massive increase of DCP5–GFP (Chicois et al, 2018) and Venus–PAT1(Zuo et al, 2022b *Preprint*) foci in hook regions (Fig 1C), all suggest that mRNA decapping is required for apical hooking.

### mRNA decay machinery targets *ASL9* for decay

**Figure 2.**
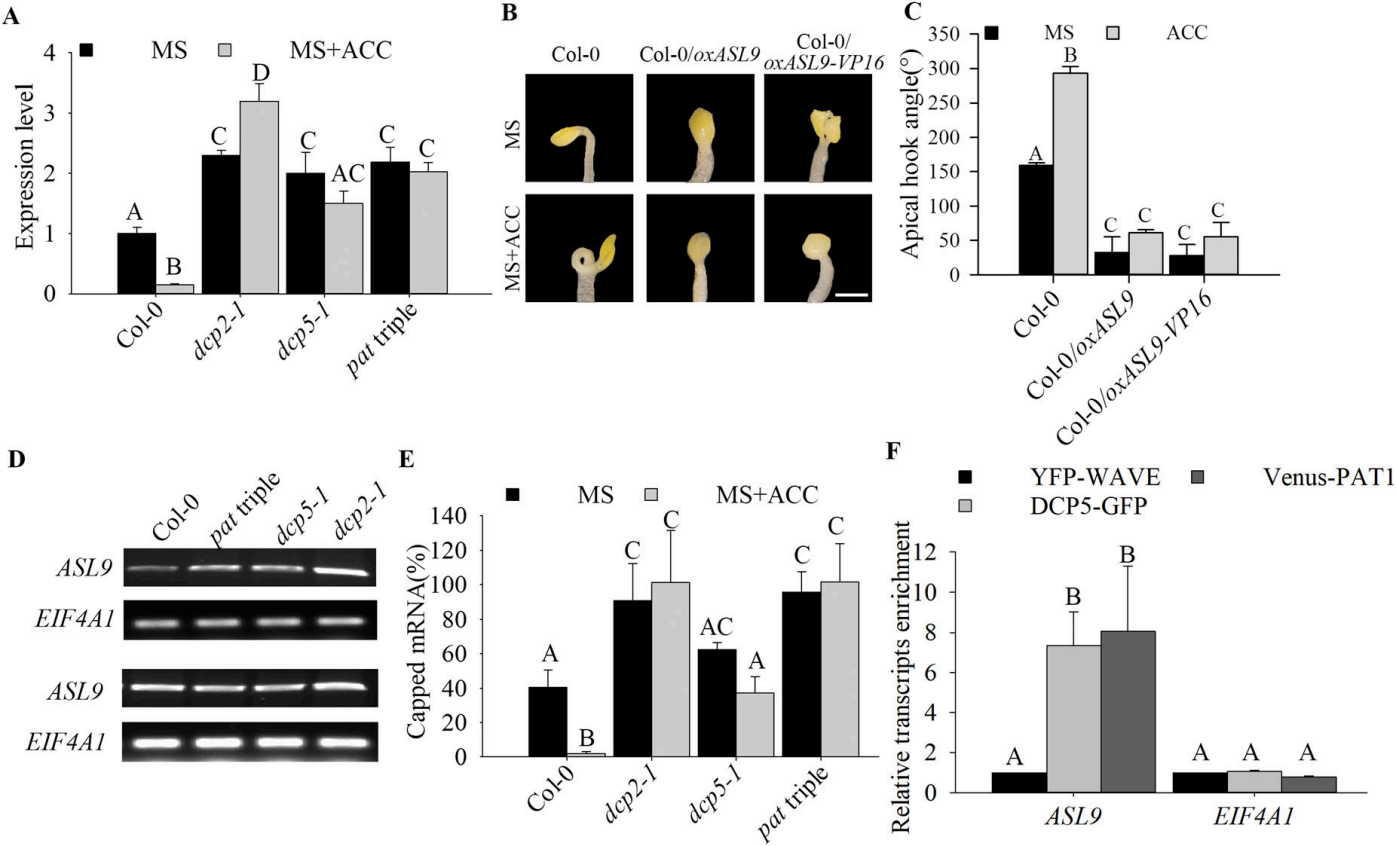
mRNA decay machinery targets *ASL9* for decay. **(A)** *ASL9* mRNA levels in cotyledons and hook regions of dark-grown Col-0, *dcp2-1*, *dcp5-1*, and *pat* triple seedlings under control or ACC treatment. Error bars indicate SE of bio-triplicates. **(B, C)** Hook phenotypes (B) and apical hook angles (C) of triple response to ACC treatment of etiolated seedlings of Col-0, Col-0/*oxASL9*, and Col-0/*oxASL9-VP16*. The experiment was repeated three times, in each repeat sample size (n)>15 for each genotype and treatment, and representative pictures are shown. The scale bar indicates 1 mm. **(D)** Accumulation of capped transcripts of *ASL9* analyzed in 4-d-old MS grown etiolated seedlings of Col-0, *pat* triple, *dcp5-1*, and *dcp2-1* by 5′-RACE-PCR. RACE-PCR products obtained using low (upper panel) and high (bottom panel) number of cycles are shown. *EIF4A1* RACE-PCR products were used as loading control. **(E)** Capped *ASL9* transcript levels using XRN1 susceptibility assay in cotyledons and hook regions of dark-grown Col-0, *dcp2-1*, *dcp5-1*, and *pat* triple seedlings. Error bars indicate SE (n = 3). **(F)** DCP5 and PAT1 bind *ASL9* transcripts. 4-d dark-grown plate seedlings with DCP5–GFP or Venus–PAT1were taken for RIP assay. *ASL9* transcript levels were normalized to those in RIP of YFP-WAVE as a non-binding control. *EIF4A1* was used as a negative control. Error bars indicate SE (n = 3). Bars marked with the same letter are not significantly different from each other (*P*-value > 0.05).

To search for transcripts responsible for the hookless phenotype, we revisited our previous RNA-seq data for *pat* triple mutant (Zuo et al, 2022b *Preprint*) and verified that transcripts of *ASL9* (*ASYMMETRIC LEAVES 2-LIKE 9*, also named *LBD3*, *LOB DOMAIN-CONTAINING PROTEIN 3*) accumulated specifically in *pat* triple mutants (Zuo et al, 2022b *Preprint*). ASL9 belongs to the large AS2/LOB (ASYMMETRIC LEAVES 2/LATERAL ORGAN BOUNDARIES) family (Matsumura et al, 2009) which includes key regulators of organ development (Xu et al, 2016). Interestingly, the ASL9 homologue ASL4 negatively regulates brassinosteroids accumulation to limit growth in organ boundaries, and overexpression of *ASL4* impairs apical hook formation and leads to dwarfed growth (Bell et al, 2012). Although *ASL4* mRNA did not accumulate in *pat* triple mutants (Zuo et al, 2022b *Preprint*), we hypothesized that ASL9 could also interfere with apical hook formation. We therefore analyzed mRNA levels of *ASL9* in ACC-treated seedlings and verified that all three sequential mRNA decapping mutants accumulated up to 30-fold higher levels of *ASL9* transcript compared with ACC-treated Col-0 seedlings (Fig 2A). Concordantly, two over-expressor lines of *ASL9* Col-0/*oxASL9* and Col-0/*oxASL9-VP16* (Naito et al, 2007) also exhibited hookless phenotypes (Fig 2B and C). However, we did not observe any changes including tighter apical hooks in *asl9-1* mutants (Fig S1C and D), suggesting other members of the AS2/LOB family act redundantly in this process. Nevertheless, these results indicate that apical hook formation in mRNA decapping deficient mutants is compromised, in part, might be due to misregulation of *ASL9*.

To determine whether *ASL9* is a target of the decapping complex, we performed 5′-RACE assays and found significantly higher levels of capped *ASL9* in mRNA decapping mutant seedlings than in Col-0 (Fig 2D). We also assayed for capped *ASL9* transcripts in ACC and mock-treated mRNA decapping mutants. By calculating the ratio between capped and total *ASL9* transcripts, we verified that with ACC treatment, mRNA decapping mutants accumulated significantly higher levels of capped *ASL9* transcripts than Col-0 (Fig 2E). Moreover, RNA immunoprecipitation (RIP) revealed enrichment of *ASL9* in DCP5–GFP and Venus–PAT1 plants compared with a MYC–YFP control line (YFP-WAVE) (Fig 2F), indicating mRNA-decapping components directly bind *ASL9* transcripts. These data confirm that *ASL9* mRNA can be found in mRNA-decapping complexes, and that mRNA decapping regulates *ASL9* mRNA levels and contributes to ACC-induced apical hook formation.

### Accumulation of *ASL9* suppresses LR formation

**Figure 3.**
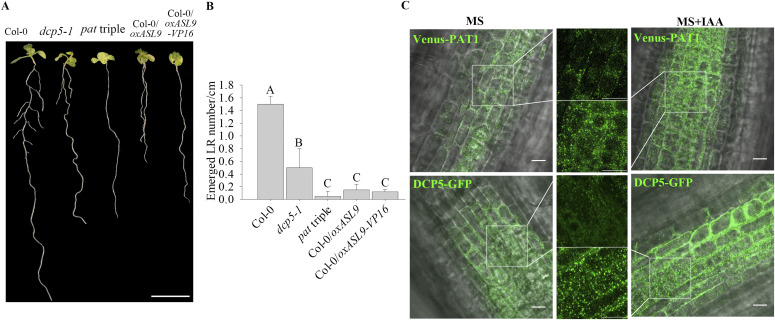
Accumulation of *ASL9* suppresses LR formation. **(A, B)** Phenotypes (A) and emerged LR density (B) of 10-d old seedlings of Col-0, *dcp5-1*, *pat* triple, Col-0/*oxASL9*, and Col-0/*oxASL9-VP16*. The experiment was repeated four times, in each repeat sample size (n)>10 for each genotype, and representative pictures are shown. The scale bar indicates 1 cm. Bars marked with the same letter are not significantly different from each other (*P*-value > 0.05). **(C)** Representative confocal microscopy pictures of root regions from 7-d old seedlings with either Venus–PAT1 or DCP5–GFP treated with MS or MS + 0.2 μM IAA for 15 min. Scale bars indicate 10 μm.

**Figure S2.**
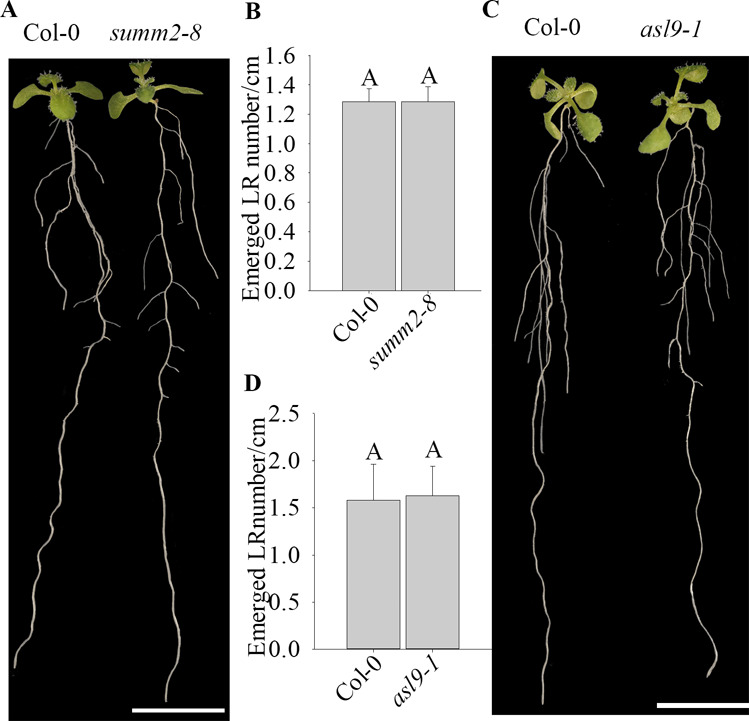
LR formation and primary root growth in *summ2-8* and *asl9-1* mutants. **(A, B)** Phenotypes (A) emerged LR density and primary root length (B) of 10-d old seedlings of Col-0 and *summ2-8*. **(C, D)** Phenotypes (C) emerged LR density and primary root length (D) of 10-d old seedlings of Col-0 and *asl9-1*. The experiment was repeated three times (n > 10), and representative pictures are shown. The scale bar indicates 1 cm. Bars marked with the same letter are not significantly different from each other (*P*-value > 0.05).

LR formation is another example of postembryonic development. In *Arabidopsis*, the first stage of LR formation requires that xylem pericyle pole cells change fate to become LR founder cells, a process positively regulated by auxin and negatively regulated by cytokinin and ethylene (Jung & McCouch, 2013; Weijers et al, 2018). We therefore examined LR formation in mRNA decapping deficient mutants *dcp5-1* and *pat* triple mutants and in both *ASL9* over-expressors and verified that LR formation was dramatically impaired in all genotypes tested (Figs 3A and B and S2A and B). However, like seen for apical hooking, *asl9-1* also appeared to display normal LR formation (Fig S2C and D). Nevertheless, LR formation defects in *dcp5-1* and *pat* triple mutants indicate that mRNA decapping is required for the commitment to LR formation. This is further substantiated by the fact that auxin application leads to a massive increase of DCP5–GFP and Venus–PAT1 foci in root regions (Fig 3C). Collectively, these data indicate mRNA decapping machinery, targeting *ASL9*, also contributes to LR formation.

### *ASL9* contributes to apical hooking and LR formation

**Figure 4.**
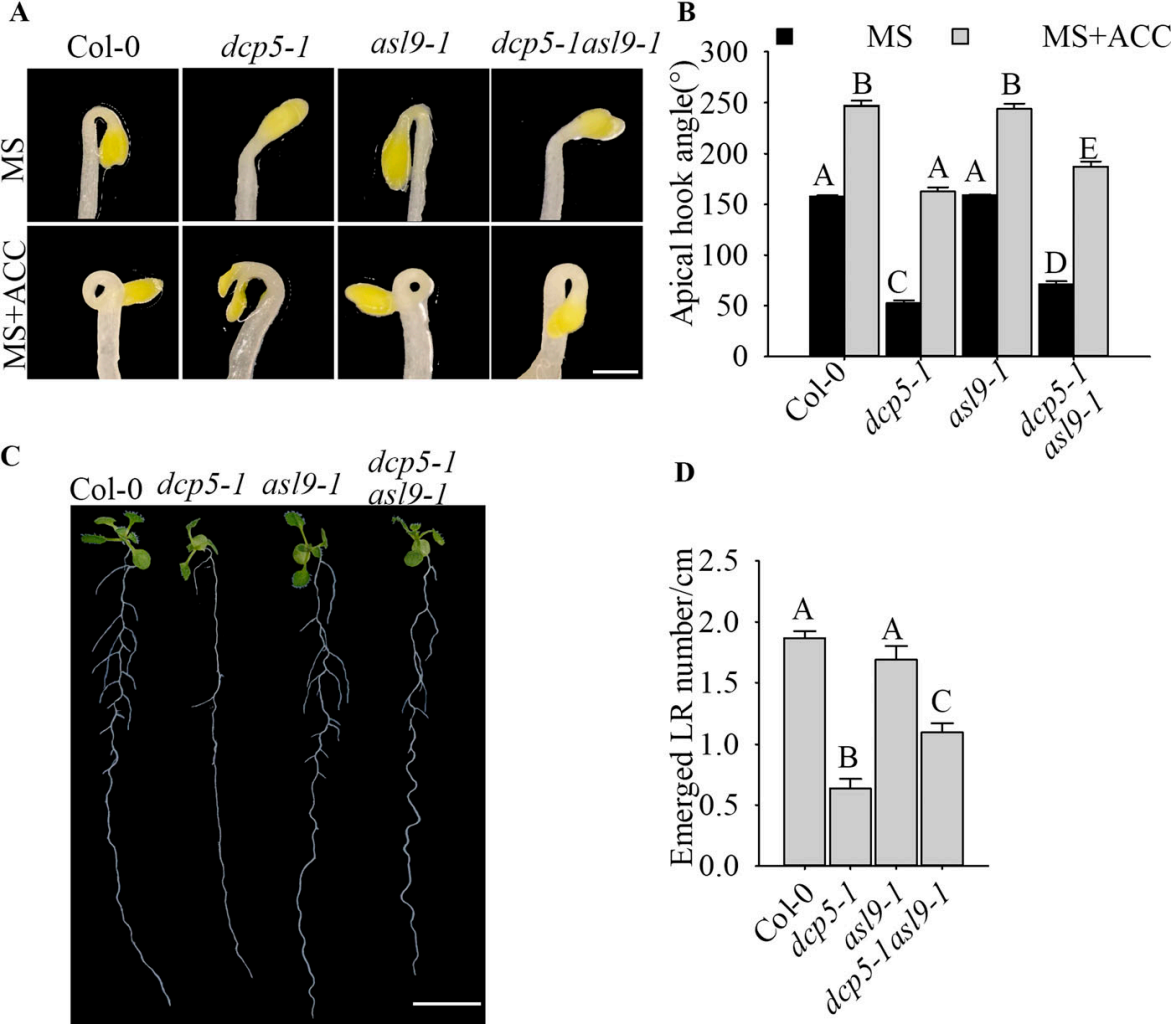
*ASL9* directly contributes to apical hooking and LR formation. **(A, B)** Hook phenotypes (A) and apical hook angles (B) in triple responses to ACC treatment of etiolated Col-0, *dcp5-1*, *asl9-1*, and *dcp5-1asl9-1* seedlings. The treatment was repeated three times, in each repeat sample size (n)>50 for each genotype and treatment, and representative pictures are shown. The scale bar indicates 1 mm. **(C, D)** Phenotypes (C) and emerged LR density (D) of 10-d old seedlings of Col-0, *dcp5-1*, *asl9-1*, and *dcp5-1asl9-1*. Treatment was repeated three times, in each repeat sample size (n)>20 for each genotype, and representative pictures are shown. The scale bar indicates 1 cm. Bars marked with the same letter are not significantly different from each other (*P*-value > 0.05).

**Figure S3.**
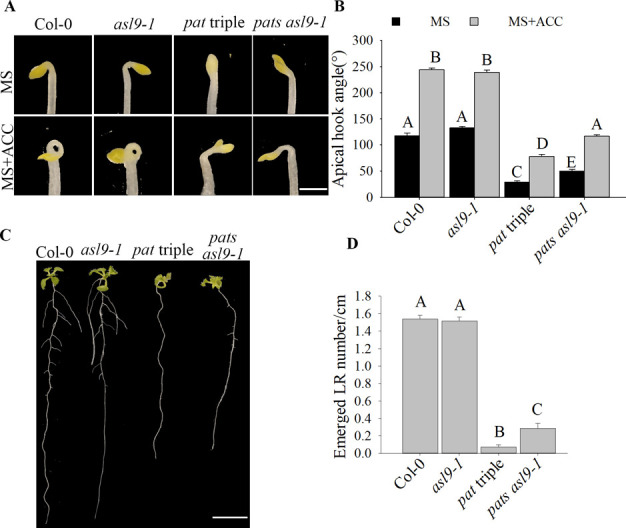
*ASL9* mutation partially restores apical hooking and LR formation phenotype in *pat* triple mutant. **(A, B)** Hook phenotypes (A) and apical hook angles (B) in triple responses to ACC treatment of etiolated Col-0, *asl9-1*, *pat* triple, and *pats asl9-1 (pat1-1path1-4path2-1summ2-8asl9-1)* seedlings. Sample size (n)>35 for each genotype and treatment, and representative pictures are shown. The scale bar indicates 1 mm. **(C, D)** Phenotypes (C) and emerged LR density (D) of 10-d old seedlings of Col-0, *asl9-1*, *pat* triple, and *pats asl9-1*. Sample size (n)>20 for each genotype, and representative pictures are shown. The scale bar indicates 1 cm. Bars marked with the same letter are not significantly different from each other (*P*-value > 0.05).

The overexpression of *ASL9* is sufficient to suppress apical hook and lateral root development. To examine more directly if *ASL9* accumulation contributes to the developmental defects in decapping mutants, we crossed *asl9-1* to both *dcp5-1* and *pat* triple mutant to generate *dcp5-1asl9-1* and *pats asl9-1* (*pat1-1path1-4path2-1summ2-8asl9-1*) mutants. We then germinated *dcp5-1asl9-1* and *pats asl9-1* seedlings in darkness in the presence or absence of ACC, and under both conditions, *dcp5-1asl9-1* and/or *pats asl9-1* made more stringent hooks than *dcp5-1* and/or *pat* triple but not as tight as Col-0 or *asl9*-1 did, indicating that the loss-of-function of *asl9* can partially suppress decapping deficient mutants hookless phenotype (Figs 4A and B and S3A and B). Moreover, the LR phenotype of *dcp5-1* and *pat* triple was also partially restored by mutating *ASL9* (Figs 4C and D and S3C and D). Thus, our data indicate that *ASL9* contributes to both apical hooking and LR development in mutant with decapping deficiencies.

### Interference with cytokinin signaling and/or exogenous auxin restores developmental defects of *ASL9* over-expressor and mRNA decay–deficient mutants

**Figure S4.**
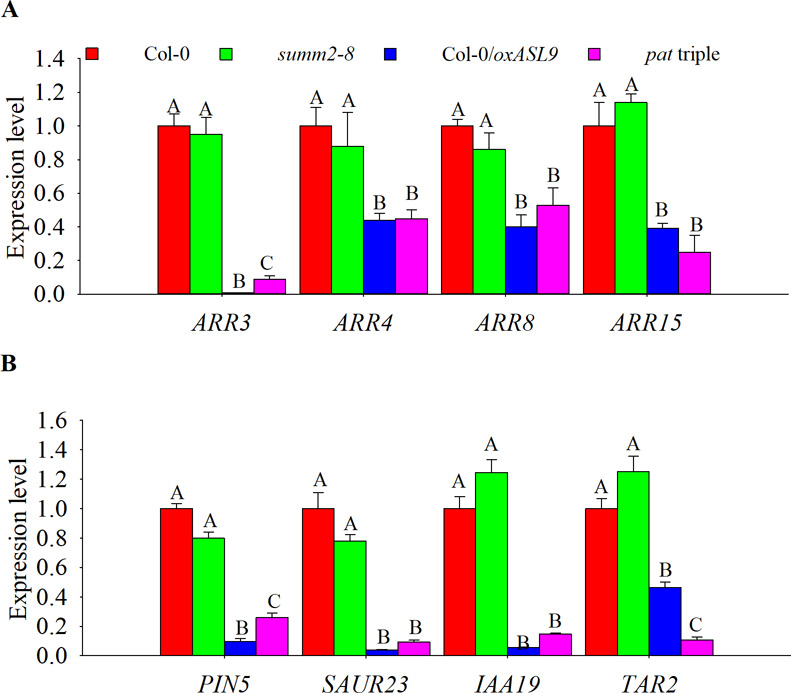
Cytokinin- and auxin-related genes expression in mRNA decay–deficient mutant and *ASL9* over-expressor. **(A, B)** Cytokinin pathway repressor genes *ARR3*, *ARR4*, *ARR8*, and *ARR15* and (B) auxin pathway genes*PIN5*, *SAUR23*, *IAA19*, *and TAR2* expression levels in 10-d-old seedlings of Col-0, *summ2-8*, Col-0/*oxASL9*, and *pat* tripe mutants. The experiment was repeated three times, error bars indicate SE of bio-triplicates. Bars marked with the same letter are not significantly different from each other (*P*-value > 0.05).

**Figure S5.**
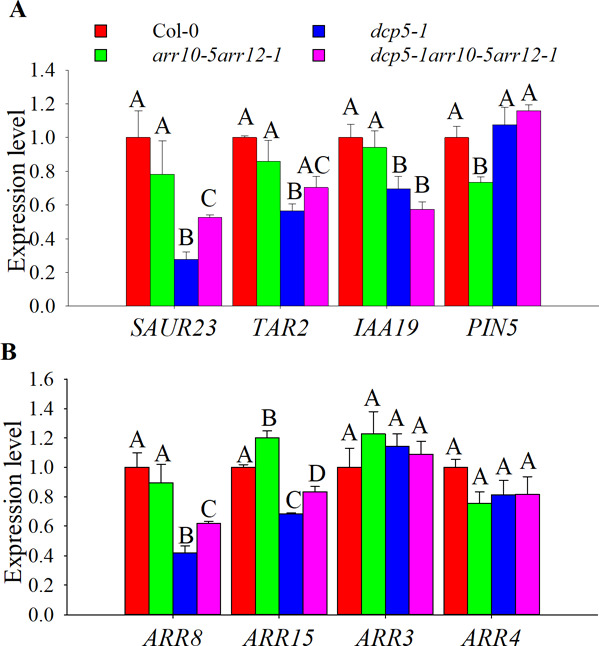
Cytokinin- and auxin-related genes expression in *dcp5-1* and *dcp5-1arr10-5arr12-1*. **(A, B)** Auxin pathway genes *SAUR23*, *TAR2*, *PIN5*, and *IAA19* and (B) cytokinin pathway repressor genes *ARR8*, *ARR15*, *ARR3*, and *ARR4* and expression levels in 10-d-old seedlings of Col-0, *arr10-5arr12-1*, *dcp5-1*, and *dcp5-1arr10-5arr12-1*. The experiment was repeated three times, error bars indicate SE of bio-triplicates. Bars marked with the same letter are not significantly different from each other (*P*-value > 0.05).

**Figure 5.**
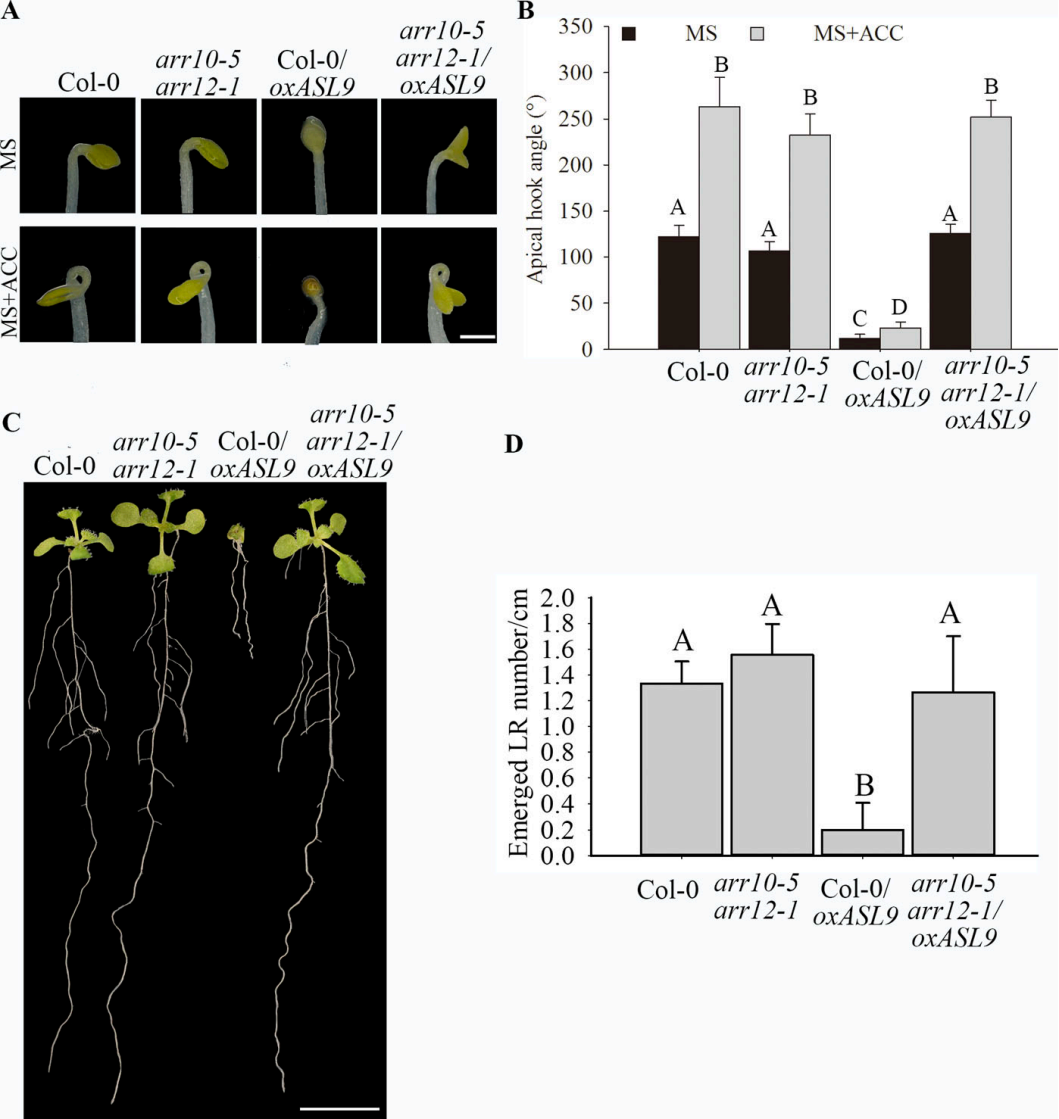
*ARR10* and *ARR12* loss-of-function restores apical hook and LR formation in *ASL9* over-expressor. **(A, B)** Hook phenotypes (A) and apical hook angles (B) in triple responses to ACC treatment of etiolated Col-0, *arr10-5arr12-1*, Col-0/*oxASL9*, and *arr10-5arr12-1/oxASL9* seedlings. The treatment was repeated three times, in each repeat sample size (n)>20 for each genotype and treatment, and representative pictures are shown. The scale bar indicates 1 mm. **(C, D)** Phenotypes (C) and emerged LR density (D) of 10-d old seedlings of Col-0, *arr10-5arr12-1*, Col-0/*oxASL9*, and *arr10-5arr12-1/oxASL9*. Treatment was repeated three times, in each repeat sample size (n)>10 for each genotype, and representative pictures are shown. The scale bar indicates 1 cm. Bars marked with the same letter are not significantly different from each other (*P*-value > 0.05).

**Figure 6.**
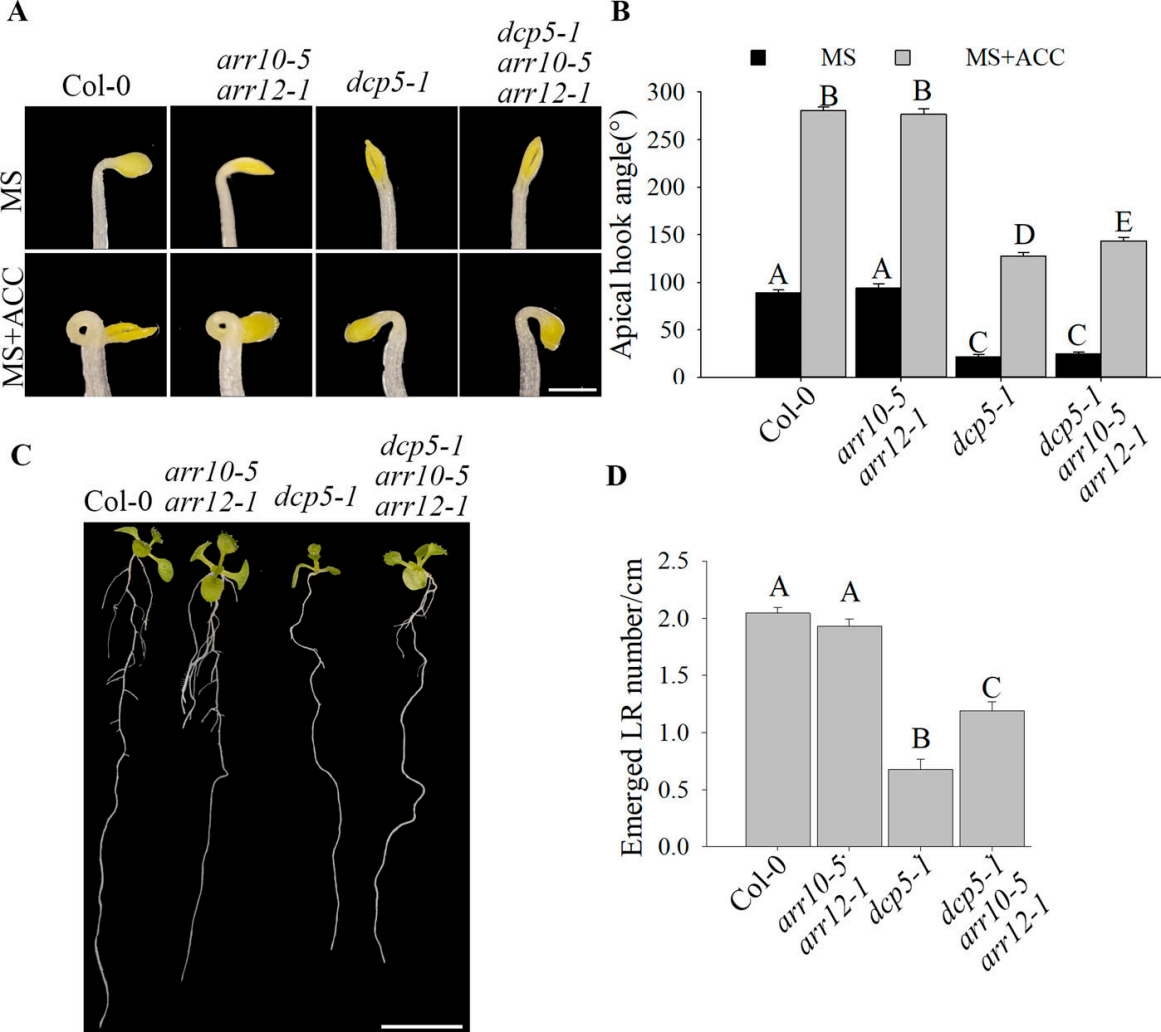
*ARR10* and *ARR12* loss-of-function partially restores apical hook and LR formation in *dcp5-1*. **(A, B)** Hook phenotypes (A) and apical hook angles (B) in triple responses to ACC treatment of etiolated Col-0, *arr10-5arr12-1*, *dcp5-1*, and *dcp5-1arr10-5arr12-1* seedlings. The treatment was repeated three times, in each repeat sample size (n)>50 for each genotype and treatment, and representative pictures are shown. The scale bar indicates 1 mm. **(C, D)** Phenotypes (C) and emerged LR density (D) of 10-d old seedlings of Col-0, *arr10-5arr12-1*, *dcp5-1*, and *dcp5-1arr10-5arr12-1*. Treatment was repeated three times, in each repeat sample size (n)>10 for each genotype, and representative pictures are shown. The scale bar indicates 1 cm. Bars marked with the same letter are not significantly different from each other (*P*-value > 0.05).

*ASL9* has been implicated in cytokinin signaling (Naito et al, 2007; Ye et al, 2021) in which ARR1, ARR10, and ARR12 are responsible for activation of cytokinin transcriptional responses (Ishida et al, 2008; Xie et al, 2018), and cytokinin acts antagonistically with auxin. Apical hooking and lateral root formation represent classic examples of auxin-dependent development (Peer et al, 2011). In support of this, *axr1* mutants showed defective apical hook formation and reduced LR numbers (Estelle & Somerville, 1987; Lehman et al, 1996). We therefore examined cytokinin- and auxin-related gene expression in both mRNA decay–deficient mutants and *ASL9* over-expressor (Figs S4 and S5). The cytokinin responsive and signaling repressors type-A ARR genes *ARR8* and *ARR15*, the auxin-induced gene *SAUR23* and the auxin biosynthesis gene *TAR2* are all repressed in these genotypes tested, which suggest a misregulation of cytokinin signaling and abrogated auxin homeostasis. To test if the developmental defects of mRNA decay mutants and Col-0/*oxASL9* are due to misregulation of cytokinin, we interfered with cytokinin pathways in *ASL9* over-expressors and decapping mutant *dcp5-1* by knocking out cytokinin-signaling activators *ARR10* and *ARR12* (Ishida et al, 2008). Interestingly, both apical hooking and LR formation phenotypes of *ASL9* over-expressors were largely restored in *arr10-5arr12-1* background (Fig 5), indicating that the developmental defects in *ASL9* over-expressors are most likely caused by misregulation of cytokinin signaling. As for *dcp5-1*, the apical hooking and LR phenotype were partially restored by mutating *arr10* and *arr12* (Fig 6), which despite not reaching the same extend as seen in *ASL9* over-expressors, was still similar to our observations in *dcp5-1asl9-1* double mutants (Figs 4 and 5). Furthermore, the expression of *ARR8*, *ARR15*, *SAUR23*, and *TAR2* in *dcp5-1* was also partially restored in *arr10-5arr12-1* background (Fig S5). Therefore, our data suggest that apical hooking and LR developmental defects in *ASL9* over-expressors and to some degree in mRNA-decapping mutants depend on functional cytokinin signaling.

**Figure S6.**
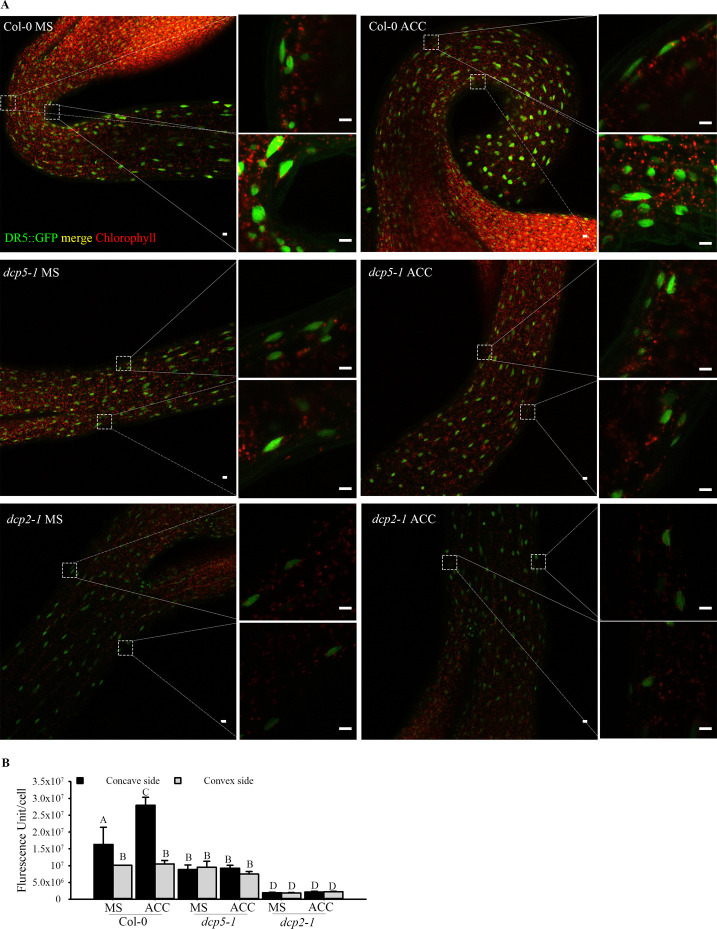
mRNA decapping mutants *dcp5-1* and *dcp2-1* exhibit repressed auxin responses in apical hook regions. **(A, B)** Representative confocal microscopy pictures (A) and quantification (B) of GFP signals in concave and convex side of apical hook regions of Col-0, *dcp5-1*, and *dcp2-1* expressed with DR5::GFP following ACC treatment. Seeds of Col-0/DR5::GFP, *dcp5-1*/DR5::GFP, and *dcp2-1*/DR5::GFP on MS or MS + ACC plates were vernalized 96 h and grown in dark for 4 d. All pictures were taken under the same confocal microscope settings. Scale bars indicate 10 µm. Bars marked with the same letter are not significantly different from each other (*P*-value > 0.05).

**Figure S7.**
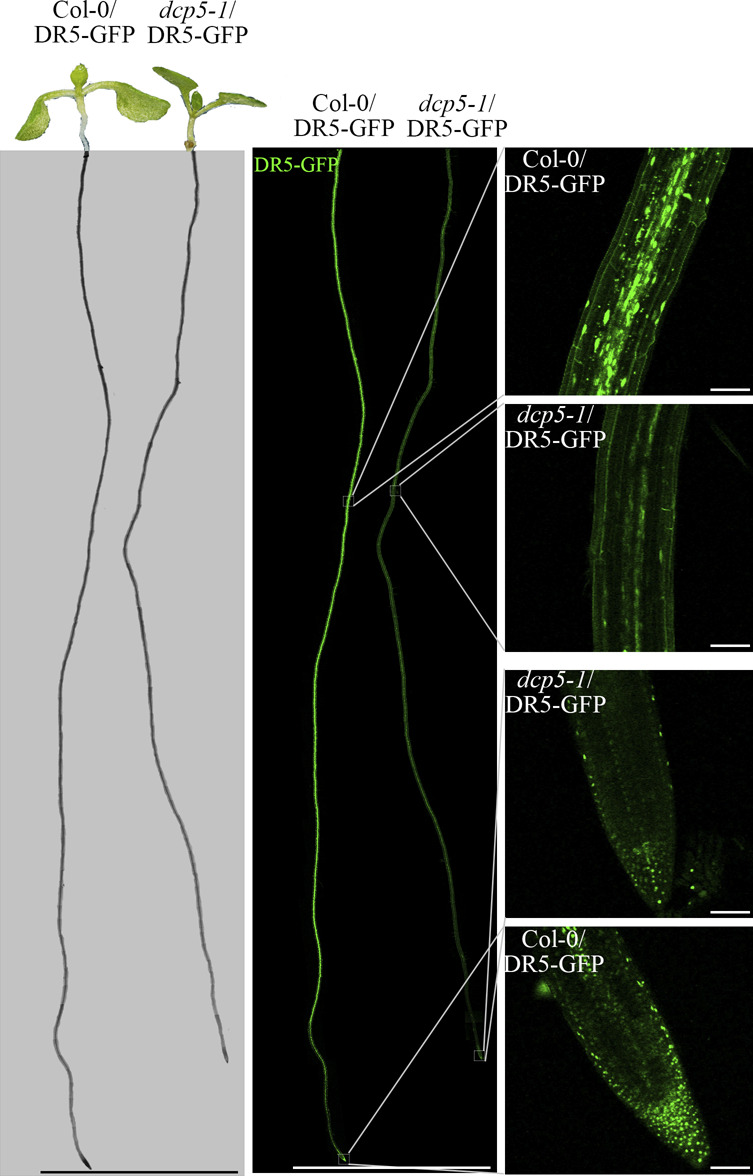
mRNA decapping mutants *dcp5-1* exhibit repressed auxin responses in root regions. Representative confocal microscopy pictures of GFP signals in root regions of 7-d old seedling of Col-0 and *dcp5-1* expressed with DR5::GFP. Seeds of Col-0/DR5::GFP and *dcp5-1*/DR5::GFP on MS plates were vernalized 96 h and grown vertically for 7 d. All pictures were taken under the same confocal microscope settings. Scale bars indicate 1 cm in the left and middle lane and 100 µm in the right lane.

**Figure S8.**
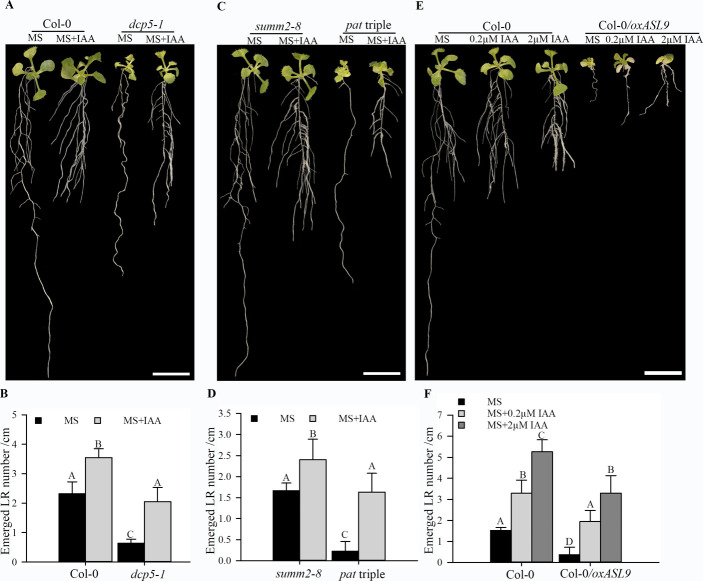
Auxin restores LR formation in mRNA decay–deficient mutants and Col-0/*oxASL9*. **(A, B)** Phenotypes (A) and emerged LR density (B) of 14-d old seedlings of *summ2-8* and *pat1-1path1-4path2-1summ2-8* on MS or MS with 0.2 µM IAA. **(C, D)** Phenotypes (C) and emerged LR density (D) of 14-d old seedlings of Col-0 and *dcp5-1* on MS or MS with 0.2 µM IAA. **(E, F)** Phenotypes (E) and emerged LR density (F) of 14-d old seedlings of Col-0 and Col-0/*oxASL9* on MS, MS with 0.2 µM IAA or MS with 2 µM IAA. Seeds on MS plates were vernalized 96 h and grown with 16/8 h light/dark at 21°C for 7 d. The seedlings were moved to MS or MS+IAA plates and grown vertically for 7 d. The treatment was repeated three times, sample size (n)>10 for each genotype and treatment, and representative pictures are shown. The scale bar indicates 1 cm. Bars marked with the same letter are not significantly different from each other (*P*-value > 0.05).

To test if repressed auxin signaling is also responsible for the developmental defects in mRNA-decapping mutants and *ASL9* over-expressors, we first confirmed the repressed auxin signaling in mRNA decay mutants by introducing the indirect auxin-responsive reporter *DR5*::GFP. We found increased GFP signals in the concave side of Col-0 apical hook region when dark-grown on MS with/without ACC but not in *dcp5-1* or *dcp2-1* under either growth condition, and the overall GFP signals in *dcp2-1* were markedly lower than Col-0 (Fig S6). We also examined DR5::GFP signal in the root area of 7-d old Col-0 and *dcp5-1* seedlings and again, overall GFP signal in *dcp5-1* were strikingly lower than Col-0 (Fig S7). Collectively, these data confirmed our supposition that repressed auxin responses in the mRNA decapping mutants affect apical hook and root developmental processes. Consistent with this notion, exogenous auxin supplementation (0.2 μM IAA) lead to partial restoration of LR formation in *dcp5-1*, *pat* triple, and Col-0/*oxASL9* (Fig S8). Collectively, our findings indicate that misregulation of cytokinin/auxin responses is partially responsible for the developmental defects in the mRNA decay mutants and *ASL9* over-expressors.

## Discussion

Developmental changes require massive overhauls of gene expression (Miyamoto et al, 2015). Apart from unlocking, effectors needed to install a new program, previous states or programs also need to be terminated (Tatapudy et al, 2017; Rodriguez et al, 2020). We report here that mRNA decay is required for certain auxin-dependent developmental processes. The stunted growth phenotype and down-regulation of developmental and auxin-responsive mRNAs in the mRNA decapping mutant (Zuo et al, 2022b *Preprint*) supports a model in which defective clearance of mRNAs hampers decision-making upon hormonal perception. Apical hooking and LR formation are classic examples of auxin-dependent developmental processes (Peer et al, 2011). In line with this, we and others observed that mRNA decay–deficient mutants are impaired in apical hooking (Fig 1) and LR formation (Fig 3) (Perea-Resa et al, 2012; Jang et al, 2019). Interestingly, among the transcripts up-regulated in these decay-deficient mutants was that of capped *ASL9/LBD3* (Fig 2), which is involved in cytokinin signaling (Naito et al, 2007). Cytokinin and auxin can act antagonistically (Su et al, 2011), and cytokinin can both attenuate apical hooking (Tantikanjana et al, 2001) and directly affect LR founder cells to prevent initiation of lateral root primordia (Laplaze et al, 2007). Our findings were that defective processing during those developmental events in mRNA decay–deficient mutants involves *ASL9* was supported by our observation that *ASL9* mRNA is directly regulated by the decapping machinery and that Col-0/*oxASL9* transgenic lines cannot reprogram to attain an apical hook or to form LRs (Figs 2 and 3), whereas loss-of-function of *asl9* partially restores the developmental defects in the decapping deficient mutants (Figs 4 and S3). In line with this, we argue that the misregulation of cytokinin-dependent and auxin-dependent signaling is partially responsible for the developmental defects in mRNA decay–deficient mutants. This is supported by the observation that auxin responses in the *dcp5-1* and *dcp2-1* mutants are repressed (Figs S6 and S7) and treating *dcp5-1*, *pat* triple, and Col-0/*oxASL9* with exogenous auxin partially restores LR formation (Fig S8). Besides misregulation of cytokinin signaling pathway in plants overexpressing *ASL9*, short-term accumulation of *ASL9* also led to down-regulation of cytokinin-responsive genes (Ye et al, 2021), indicating a negative role of *ASL9* in regulating cytokinin responses. However, the fact that the developmental defects in *ASL9* over-expressors are largely restored by knocking out two cytokinin signaling activator genes *ARR10* and *ARR12* suggest the function of *ASL9* during apical hooking, and LR formation largely depends on *ARR10* and *ARR12*. In line with this, the developmental defects of *dcp5-1* are also partially restored in *asl9-1* and *arr10-5arr12-1* backgrounds (Figs 4 and 6), but in addition to *ASL9* and *ARR*s, other unidentified factors also contribute to the defects in apical hook and LR formation in decapping mutants.

Arabidopsis contains 42 *LBD/ASL* genes (Matsumura et al, 2009), among these genes, *LBD16*, *LBD17*, *LBD18*, and *LBD29* control lateral roots formation and regulate plant regeneration (Fan et al, 2012), and overexpression of another member *ASL4* also impairs apical hook (Bell et al, 2012). The partial restoration of apical hooking and LR formation caused by *asl9* mutation in *dcp5-1* and *pat* triple mutant (Figs 4 and S3) suggest that other *ASL*s and/or non-*ASL* genes also contribute to the developmental defects in decapping mutants. Besides lateral root formation, it was recently reported that *Arabidopsis* LBD3, together with LBD4, functions as rate-limiting components in activating and promoting root secondary growth, which is also tightly regulated by auxin and cytokinin, indicating that LBDs balance primary and secondary root growth (Smetana et al, 2019; Smith et al, 2020; Xiao et al, 2020; Ye et al, 2021). Together with auxin, cytokinin plays crucial roles in vascular development through the two-component signaling system, and plants with mutations in cytokinin receptor or type B-*ARRs* exhibit vasculature defects (Mähönen et al, 2006; Yokoyama 2007; Kondo et al, 2011). Hence, we cannot exclude the possibility that the developmental defects we observed in mRNA decapping mutants and *ASL9* over-expressors are also related to their vascular development.

Deadenylated mRNA can be degraded via either 3′–5′ exosomal exonucleases and SOV/DIS3L2 or via the 5′–3′ XRN activity of the decapping complex (Garneau et al, 2007; Sorenson et al, 2018). Sorenson et al (2018) found that *ASL9* expression is dependent on both VCS and SOV based on their transcriptome analysis, so that *ASL9* might be a target of both pathways (Sorenson et al, 2018). Although more direct data are needed to conclude whether SOV can directly regulate *ASL9* mRNA levels, we have shown that *ASL9* is a target of the mRNA decapping machinery. However, because the Col-0 accession is a *sov* mutant and has no developmental defects, the SOV decay pathway probably only plays an accessory role in regulation of *ASL9*. The function of PBs in mRNA regulation has been controversial because mRNAs in PBs may be sequestered for degradation or re-enter polysomal translation complexes (Franks & Lykke-Andersen, 2008). Yeast PAT1 has also been found to repress translation (Coller & Parker, 2005), and a recent study has confirmed that PBs function as mRNA reservoirs in dark-grown *Arabidopsis* seedlings (Jang et al, 2019). These data open the possibility that *ASL9* might be also regulated at the translational level by the decapping machinery. Nevertheless, our finding of direct interaction of *ASL9* transcripts with DCP5 and PAT1, together with the accumulation of capped *ASL9* in mRNA decay mutants, indicates that *ASL9* misregulation in *dcp2-1*, *dcp5-1*, and *pat* triple mutants is due to mRNA decapping deficiency (Fig 2).

## Materials and Methods

### Plant materials and growth conditions

*Arabidopsis thaliana* ecotype Columbia (Col-0) was used as a control. All mutants used in this study are listed in Table S1. T-DNA insertion lines for AT5G13570 (*DCP2*) *dcp2-1* (Salk_000519), At1g26110 (*DCP5*) *dcp5-1* (Salk_008881), and double mutant *arr10-5arr12-1* have been described (Xu et al, 2006; Ishida et al, 2008; Xu & Chua, 2009). The T-DNA line for AT1g16530 (*ASL9*) is SAIL_659_D08 with insertion in the first exon. Primers for newly described T-DNA lines are provided in Table S2. *pat* triple mutant, Venus–PAT1, and DCP5–GFP transgenic lines have also been described (Chicois et al, 2018; Zuo et al, 2022b *Preprint*). The YFP-WAVE line was from NASC (Geldner et al, 2009). Col-0/*oxASL9* line has been described before (Naito et al, 2007).

Table S1. Mutants used in this study.

Table S2. Primers used in this study.

Plants were grown in 9 × 9 cm or 4 × 5 cm pots at 21°C with 8/16 h light/dark regime, or on plates containing Murashige–Skoog (MS) salts medium (Duchefa), 1% sucrose, and 1% agar with 16/8 h light/dark.

### Plant treatments

For ethylene triple response assays, seeds were plated on normal MS and MS + 50 µM ACC, vernalized 96 h, and placed in the dark at 21°C for 4 d before pictures were taken. Apical hook angle is defined as 180° minus the angle between the tangential of the apical part with the axis of the lower part of the hypocotyl, in the case of hook exaggeration, 180° plus that angle is defined as the angle of hook curvature (Vandenbussche et al, 2010). Cotyledon and hook regions of etiolated seedlings were collected after placing in the dark at 21°C for 4 d for gene expression and XRN1 assay. For LR formation assays, seeds on MS plates were vernalized 96 h and grown with 16/8 h light/dark at 21°C vertically for 10 d. For external IAA application for LR formation experiments, seeds on MS plates were vernalized 96 h and grown with 16/8 h light/dark at 21°C for 7 d and the seedlings were moved to MS or MS+IAA plates and grown vertically for 7 d.

### Cloning and transgenic lines

pGreenIIM DR5V2-ntdtomato/DR5-n3GFP has been published previously (Liao et al, 2015). Arabidopsis transformation was performed by floral dipping (Clough & Bent, 1998) for Col-0/DR5::GFP and thereafter Col-0/DR5::GFP was crossed to *dcp5-1* and *dcp2-1*^*het*^ to achieve *dcp5-1*/DR5::GFP and *dcp2-1*/DR5::GFP. *arr10-5arr12-1/oxASL9* was generated by vacuum infiltrating *arr10-5arr12-1* with *A. tumefaciens* strain EHA101 harbouring pSK1-ASL9 (Naito et al, 2007). Transformants were selected on hygromycin (30 mg/l) or methotrexate (0.1 mg/l) MS agar, and survivors were tested for transcript expression by qRT-PCR and protein expression by immuno-blotting and at least two independent lines were used for further analysis.

### Protein extraction, SDS–PAGE, and immunoblotting

Tissue was ground in liquid nitrogen and 4 × SDS buffer (Novex) was added and heated at 95°C for 5 min, cooled to room temperature for 10 min, samples were centrifuged 5 min at 15,682*g*. Supernatants were separated on 10% SDS–PAGE gels, electroblotted to PVDF membrane (GE Healthcare), blocked in 5% (wt/vol) milk in TBS-Tween 20 (0.1%, vol/vol), and incubated 1 h to overnight with primary antibodies (anti-GFP [1:5,000; AMS Biotechnology]). Membranes were washed 3 × 10 min in TBS-T (0.1%) before 1 h incubation in secondary antibodies (anti-rabbit HRP or AP conjugate [1:5,000; Promega]). Chemiluminescent substrate (ECL Plus, Pierce) was applied before camera detection. For AP-conjugated primary antibodies, membranes were incubated in NBT/BCIP (Roche) until bands were visible.

### Confocal microscopy

Imaging was performed using a Zeiss LSM 700 confocal microscope. The confocal images were analyzed with Zen2012 (Zeiss) and ImageJ software. Representative maximum intensity projection images of 10 Z-stacks per image have been shown in Figs 1, 3, S5, and S6.

### RNA extraction and qRT-PCR

Total RNA from tissues was extracted with TRIzol Reagent (Thermo Fisher Scientific), 2 µg total RNA were treated with DNAse I (Thermo Fisher Scientific), and reverse transcribed into cDNA using RevertAid First Strand cDNA Synthesis Kit according to the manufacturer’s instructions (Thermo Fisher Scientific). The *ACT2* gene was used as an internal control. qRT-PCR analysis was performed on a Bio-Rad CFX96 system with SYBR Green Master Mix (Thermo Fisher Scientific). Primers are listed in Table S2. All experiments were repeated at least three times each in technical triplicates.

### In vitro XRN1 susceptibility assay

Transcripts XRN1 susceptibility was determined as described (Mukherjee et al, 2012; Kiss et al, 2016) with some modification. Total RNA was extracted from tissues using the NucleoSpin RNA Plant kit (Machery-Nagel). 1 µg RNA was incubated with either 1 unit of XRN1 (New England Biolabs) or water for 2 h at 37°C, loss of ribosomal RNA bands on gel electrophoresis was used to ensure XRN1 efficiency, after heating inactivation under 70°C for 10 min, half of the digest was then reverse transcribed into random primed cDNA with RevertAid First Strand cDNA Synthesis Kit (Thermo Fisher Scientific). Capped target transcript accumulation was measured by comparing transcript levels from XRN1-treated versus mock-treated samples using qRT-PCR (*EIF4A1* serves as inner control) for the individual genotypes (Mukherjee et al, 2012; Roux et al, 2015; Kiss et al, 2016).

### RIP assay

RIP was performed as previously described (Streitner et al, 2012). 1.5 g tissues were fixated by vacuum infiltration with 1% formaldehyde for 20 min followed by 125 mM glycine for 5 min. Tissues were ground in liquid nitrogen and RIP lysis buffer (50 mM Tris–HCl, pH 7.5; 150 mM NaCl; 4 mM MgCl2; 0.1% Igepal; 5 mM DTT; 100 U/ml Ribolock [Thermo Fisher Scientific]; 1 mM PMSF; protease inhibitor cocktail [Roche]) was added at 1.5 ml/g tissue powder. Following 15 min centrifugation at 4°C and 15,682*g*, supernatants were incubated with GFP-Trap A beads (ChromoTek) for 4 h at 4°C. Beads were washed three times with buffer (50 mM Tris–HCl, pH 7.5; 500 mM NaCl; 4 mM MgCl2; 0.5% Igepal; 0.5% sodium deoxycholate; 0.1% SDS; 2 M urea; 2 mM DTT before RNA extraction with TRIzol reagent [Thermo Fisher Scientific]). Transcript levels in input and IP samples were quantified by qRT-PCR, and levels in IP samples were corrected with their own input values and then normalized to YFP-WAVE lines for enrichment.

### 5′-RACE assay

5′-RACE assay was performed using the FirstChoice RLM-RACE kit (Thermo Fisher Scientific) following manufacture’s instruction. RNAs were extracted from 4-d-old etiolated seedlings with the NucleoSpin RNA Plant kit (Machery-Nagel), and PCRs were performed using a low (26–28) or high (30–32) number of cycles. Specific primers for the 5′ RACE adapter and for the genes tested are listed in Table S2.

### Statistical analysis

Statistical details of experiments are reported in the figures and legends. Systat software was used for data analysis. Statistical significance between groups was determined by one-way ANOVA (analysis of variance) followed by Holm–Sidak test.

## Supplementary Material

Reviewer comments

## References

[bib1] Balagopal V, Parker R (2009) Polysomes, P bodies and stress granules: States and fates of eukaryotic mRNAs. Curr Opin Cell Biol 21: 403–408. 10.1016/j.ceb.2009.03.005 19394210 PMC2740377

[bib2] Bell EM, Lin WC, Husbands AY, Yu LF, Jaganatha V, Jablonska B, Mangeon A, Neff MM, Girke T, Springer PS (2012) Arabidopsis lateral organ boundaries negatively regulates brassinosteroid accumulation to limit growth in organ boundaries. Proc Natl Acad Sci U S A 109: 21146–21151. 10.1073/pnas.1210789109 23213252 PMC3529045

[bib4] Bleecker AB, Estelle MA, Somerville C, Kende H (1988) Insensitivity to ethylene conferred by a dominant mutation in Arabidopsis thaliana. Science 241: 1086–1089. 10.1126/science.241.4869.1086 17747490

[bib5] Bonnerot C, Boeck R, Lapeyre B (2000) The two proteins Pat1p (Mrt1p) and Spb8p interact in vivo, are required for mRNA decay, and are functionally linked to Pab1p. Mol Cell Biol 20: 5939–5946. 10.1128/mcb.20.16.5939-5946.2000 10913177 PMC86071

[bib6] Brengues M, Teixeira D, Parker R (2005) Movement of eukaryotic mRNAs between polysomes and cytoplasmic processing bodies. Science 310: 486–489. 10.1126/science.1115791 16141371 PMC1863069

[bib8] Charenton C, Gaudon-Plesse C, Fourati Z, Taverniti V, Back R, Kolesnikova O, Seraphin B, Graille M (2017) A unique surface on Pat1 C-terminal domain directly interacts with Dcp2 decapping enzyme and Xrn1 5′-3′ mRNA exonuclease in yeast. Proc Natl Acad Sci U S A 114: E9493–E9501. 10.1073/pnas.1711680114 29078363 PMC5692570

[bib9] Chaudhury AM, Letham S, Craig S, Dennis ES (1993) Amp1 - a mutant with high cytokinin levels and altered embryonic pattern, faster vegetative growth, constitutive photomorphogenesis and precocious flowering. Plant J 4: 907–916. 10.1046/j.1365-313x.1993.04060907.x

[bib10] Chicois C, Scheer H, Garcia S, Zuber H, Mutterer J, Chicher J, Hammann P, Gagliardi D, Garcia D (2018) The UPF1 interactome reveals interaction networks between RNA degradation and translation repression factors in Arabidopsis. Plant J 96: 119–132. 10.1111/tpj.14022 29983000

[bib11] Chowdhury A, Kalurupalle S, Tharun S (2014) Pat1 contributes to the RNA binding activity of the Lsm1-7-Pat1 complex. RNA 20: 1465–1475. 10.1261/rna.045252.114 25035297 PMC4138329

[bib12] Clough SJ, Bent AF (1998) Floral dip: A simplified method for agrobacterium-mediated transformation of Arabidopsis thaliana. Plant J 16: 735–743. 10.1046/j.1365-313x.1998.00343.x 10069079

[bib13] Coller J, Parker R (2005) General translational repression by activators of mRNA decapping. Cell 122: 875–886. 10.1016/j.cell.2005.07.012 16179257 PMC1853273

[bib14] Crisp PA, Ganguly D, Eichten SR, Borevitz JO, Pogson BJ (2016) Reconsidering plant memory: Intersections between stress recovery, RNA turnover, and epigenetics. Sci Adv 2: e1501340. 10.1126/sciadv.1501340 26989783 PMC4788475

[bib15] Crisp PA, Ganguly DR, Smith AB, Murray KD, Estavillo GM, Searle I, Ford E, Bogdanovic O, Lister R, Borevitz JO, (2017) Rapid recovery gene downregulation during excess-light stress and recovery in Arabidopsis. Plant Cell 29: 1836–1863. 10.1105/tpc.16.00828 28705956 PMC5590493

[bib17] Essig K, Kronbeck N, Guimaraes JC, Lohs C, Schlundt A, Hoffmann A, Behrens G, Brenner S, Kowalska J, Lopez-Rodriguez C, (2018) Roquin targets mRNAs in a 3′-UTR-specific manner by different modes of regulation. Nat Commun 9: 3810. 10.1038/s41467-018-06184-3 30232334 PMC6145892

[bib18] Estelle MA, Somerville C (1987) Auxin-resistant mutants of arabidopsis-thaliana with an altered morphology. Mol Gen Genet 206: 200–206. 10.1007/bf00333575

[bib19] Fan MZ, Xu CY, Xu K, Hu YX (2012) Lateral organ boundaries domain transcription factors direct callus formation in Arabidopsis regeneration. Cell Res 22: 1169–1180. 10.1038/cr.2012.63 22508267 PMC3391013

[bib20] Franks TM, Lykke-Andersen J (2008) The control of mRNA decapping and P-body formation. Mol Cell 32: 605–615. 10.1016/j.molcel.2008.11.001 19061636 PMC2630519

[bib21] Garneau NL, Wilusz J, Wilusz CJ (2007) The highways and byways of mRNA decay. Nat Rev Mol Cell Biol 8: 113–126. 10.1038/nrm2104 17245413

[bib22] Geldner N, Denervaud-Tendon V, Hyman DL, Mayer U, Stierhof YD, Chory J (2009) Rapid, combinatorial analysis of membrane compartments in intact plants with a multicolor marker set. Plant J 59: 169–178. 10.1111/j.1365-313x.2009.03851.x 19309456 PMC4854200

[bib23] Gerstberger S, Hafner M, Tuschl T (2014) A census of human RNA-binding proteins. Nat Rev Genet 15: 829–845. 10.1038/nrg3813 25365966 PMC11148870

[bib24] Gloggnitzer J, Akimcheva S, Srinivasan A, Kusenda B, Riehs N, Stampfl H, Bautor J, Dekrout B, Jonak C, Jimenez-Gomez JM, (2014) Nonsense-mediated mRNA decay modulates immune receptor levels to regulate plant antibacterial defense. Cell Host Microbe 16: 376–390. 10.1016/j.chom.2014.08.010 25211079

[bib25] Guzman P, Ecker JR (1990) Exploiting the triple response of Arabidopsis to identify ethylene-related mutants. Plant Cell 2: 513–523. 10.2307/3869113 2152173 PMC159907

[bib26] Hu YM, Vandenbussche F, Van Der Straeten D (2017) Regulation of seedling growth by ethylene and the ethylene-auxin crosstalk. Planta 245: 467–489. 10.1007/s00425-017-2651-6 28188422

[bib27] Ishida K, Yamashino T, Yokoyama A, Mizuno T (2008) Three type-B response regulators, ARR1, ARR10 and ARR12, play essential but redundant roles in cytokinin signal transduction throughout the life cycle of Arabidopsis thaliana. Plant Cell Physiol 49: 47–57. 10.1093/pcp/pcm165 18037673

[bib28] Jang GJ, Yang JY, Hsieh HL, Wu SH (2019) Processing bodies control the selective translation for optimal development of Arabidopsis young seedlings. Proc Natl Acad Sci U S A 116: 6451–6456. 10.1073/pnas.1900084116 30850529 PMC6442596

[bib29] Jing HW, Strader LC (2019) Interplay of auxin and cytokinin in lateral root development. Int J Mol Sci 20: 486. 10.3390/ijms20030486 30678102 PMC6387363

[bib30] Jung JKH, McCouch S (2013) Getting to the roots of it: Genetic and hormonal control of root architecture. Front Plant Sci 4: 186. 10.3389/fpls.2013.00186 23785372 PMC3685011

[bib32] Kiss DL, Oman KM, Dougherty JA, Mukherjee C, Bundschuh R, Schoenberg DR (2016) Cap homeostasis is independent of poly(A) tail length. Nucleic Acids Res 44: 304–314. 10.1093/nar/gkv1460 26673707 PMC4705677

[bib33] Kondo Y, Hirakawa Y, Kieber JJ, Fukuda H (2011) CLE peptides can negatively regulate protoxylem vessel formation via cytokinin signaling. Plant Cell Physiol 52: 37–48. 10.1093/pcp/pcq129 20802224 PMC3023848

[bib34] Laplaze L, Benkova E, Casimiro I, Maes L, Vanneste S, Swarup R, Weijers D, Calvo V, Parizot B, Herrera-Rodriguez MB, (2007) Cytokinins act directly on lateral root founder cells to inhibit root initiation. Plant Cell 19: 3889–3900. 10.1105/tpc.107.055863 18065686 PMC2217640

[bib35] Lehman A, Black R, Ecker JR (1996) HOOKLESS1, an ethylene response gene, is required for differential cell elongation in the Arabidopsis hypocotyl. Cell 85: 183–194. 10.1016/s0092-8674(00)81095-8 8612271

[bib36] Li X, Mo XR, Shou HX, Wu P (2006) Cytokinin-mediated cell cycling arrest of pericycle founder cells in lateral root initiation of Arabidopsis. Plant Cell Physiol 47: 1112–1123. 10.1093/pcp/pcj082 16854941

[bib37] Liao CY, Smet W, Brunoud G, Yoshida S, Vernoux T, Weijers D (2015) Reporters for sensitive and quantitative measurement of auxin response. Nat Methods 12: 207–210. 10.1038/nmeth1115-1098a 25643149 PMC4344836

[bib38] Lobel JH, Tibble RW, Gross JD (2019) Pat1 activates late steps in mRNA decay by multiple mechanisms. Proc Natl Acad Sci U S A 116: 23512–23517. 10.1073/pnas.1905455116 31690658 PMC6876151

[bib39] Mähönen AP, Bishopp A, Higuchi M, Nieminen KM, Kinoshita K, Törmäkangas K, Ikeda Y, Oka A, Kakimoto T, Helariutta Y (2006) Cytokinin signaling and its inhibitor AHP6 regulate cell fate during vascular development. Science 311: 94–98. 10.1126/science.1118875 16400151

[bib41] Matsumura Y, Iwakawa H, Machida Y, Machida C (2009) Characterization of genes in the ASYMMETRIC LEAVES_2_/LATERAL ORGAN BOUNDARIES (AS2/LOB) family in Arabidopsis thaliana, and functional and molecular comparisons between AS2 and other family members. Plant J 58: 525–537. 10.1111/j.1365-313x.2009.03797.x 19154202 PMC2721968

[bib42] Merret R, Descombin J, Juan YT, Favory JJ, Carpentier MC, Chaparro C, Charng YY, Deragon JM, Bousquet-Antonelli C (2013) XRN4 and LARP1 are required for a heat-triggered mRNA decay pathway involved in plant acclimation and survival during thermal stress. Cell Rep 5: 1279–1293. 10.1016/j.celrep.2013.11.019 24332370

[bib43] Miyamoto T, Furusawa C, Kaneko K (2015) Pluripotency, differentiation, and reprogramming: A gene expression dynamics model with epigenetic feedback regulation. PLoS Comput Biol 11: e1004476. 10.1371/journal.pcbi.1004476 26308610 PMC4550282

[bib82] Mukherjee C, Patil DP, Kennedy BA, Bakthavachalu B, Bundschuh R, Schoenberg DR (2012) Identification of cytoplasmic capping targets reveals a role for cap homeostasis in translation and mRNA stability. Cell Rep 2: 674–684. 10.1016/j.celrep.2012.07.011 22921400 PMC3462258

[bib45] Naito T, Yamashino T, Kiba T, Koizumi N, Kojima M, Sakakibara H, Mizuno T (2007) A link between cytokinin and ASL9 (ASYMMETRIC LEAVES 2 LIKE 9) that belongs to the AS2/LOB (LATERAL ORGAN BOUNDARIES) family genes in Arabidopsis thaliana. Biosci Biotechnol Biochem 71: 1269–1278. 10.1271/bbb.60681 17485849

[bib47] Newman R, Ahlfors H, Saveliev A, Galloway A, Hodson DJ, Williams R, Besra GS, Cook CN, Cunningham AF, Bell SE, (2017) Maintenance of the marginal-zone B cell compartment specifically requires the RNA-binding protein ZFP36L1. Nat Immunol 18: 683–693. 10.1038/ni.3724 28394372 PMC5438597

[bib48] Ozgur S, Chekulaeva M, Stoecklin G (2010) Human Pat1b connects deadenylation with mRNA decapping and controls the assembly of processing bodies. Mol Cell Biol 30: 4308–4323. 10.1128/mcb.00429-10 20584987 PMC2937563

[bib49] Peer WA, Blakeslee JJ, Yang HB, Murphy AS (2011) Seven things we think we know about auxin transport. Mol Plant 4: 487–504. 10.1093/mp/ssr034 21505044

[bib50] Perea-Resa C, Hernandez-Verdeja T, Lopez-Cobollo R, Castellano MD, Salinas J (2012) LSM proteins provide accurate splicing and decay of selected transcripts to ensure normal Arabidopsis development. Plant Cell 24: 4930–4947. 10.1105/tpc.112.103697 23221597 PMC3556967

[bib51] Perea-Resa C, Carrasco-Lopez C, Catala R, Tureckova V, Novak O, Zhang WP, Sieburth L, Jimenez-Gomez JM, Salinas J (2016) The LSM1-7 complex differentially regulates Arabidopsis tolerance to abiotic stress conditions by promoting selective mRNA decapping. Plant Cell 28: 505–520. 10.1105/tpc.15.00867 26764377 PMC4790874

[bib52] Petersen M, Brodersen P, Naested H, Andreasson E, Lindhart U, Johansen B, Nielsen HB, Lacy M, Austin MJ, Parker JE, (2000) Arabidopsis MAP kinase 4 negatively regulates systemic acquired resistance. Cell 103: 1111–1120. 10.1016/s0092-8674(00)00213-0 11163186

[bib53] Riefler M, Novak O, Strnad M, Schmülling T (2005) Arabidopsis cytokinin receptor mutants reveal functions in shoot growth, leaf senescence, seed size, germination, root development, and cytokinin metabolism. Plant Cell 18: 40–54. 10.1105/tpc.105.037796 16361392 PMC1323483

[bib54] Riehs-Kearnan N, Gloggnitzer J, Dekrout B, Jonak C, Riha K (2012) Aberrant growth and lethality of Arabidopsis deficient in nonsense-mediated RNA decay factors is caused by autoimmune-like response. Nucleic Acids Res 40: 5615–5624. 10.1093/nar/gks195 22379136 PMC3384318

[bib55] Rodriguez E, Chevalier J, Olsen J, Ansbol J, Kapousidou V, Zuo Z, Svenning S, Loefke C, Koemeda S, Drozdowskyj PS, (2020) Autophagy mediates temporary reprogramming and dedifferentiation in plant somatic cells. EMBO J 39: e103315. 10.15252/embj.2019103315 31930531 PMC7024839

[bib56] Roux ME, Rasmussen MW, Palma K, Lolle S, Regue AM, Bethke G, Glazebrook J, Zhang WP, Sieburth L, Larsen MR, (2015) The mRNA decay factor PAT1 functions in a pathway including MAP kinase 4 and immune receptor SUMM2. EMBO J 34: 593–608. 10.15252/embj.201488645 25603932 PMC4365030

[bib57] Schaller GE, Bishopp A, Kieber JJ (2015) The yin-yang of hormones: Cytokinin and auxin interactions in plant development. Plant Cell 27: 44–63. 10.1105/tpc.114.133595 25604447 PMC4330578

[bib58] Smetana O, Mäkilä R, Lyu M, Amiryousefi A, Sánchez Rodríguez F, Wu M, Solé-Gil A, Leal Gavarrón M, Siligato R, Miyashima S, (2019) High levels of auxin signalling define the stem-cell organizer of the vascular cambium. Nature 565: 485–489. 10.1038/s41586-018-0837-0 30626967

[bib59] Smit ME, McGregor SR, Sun H, Gough C, Bågman AM, Soyars CL, Kroon JT, Gaudinier A, Williams CJ, Yang X, (2020) A PXY-mediated transcriptional network integrates signaling mechanisms to control vascular development in Arabidopsis. Plant Cell 32: 319–335. 10.1105/tpc.19.00562 31806676 PMC7008486

[bib60] Sorenson RS, Deshotel MJ, Johnson K, Adler FR, Sieburth LE (2018) Arabidopsis mRNA decay landscape arises from specialized RNA decay substrates, decapping- mediated feedback, and redundancy. Proc Natl Acad Sci U S A 115: E1485–E1494. 10.1073/pnas.1712312115 29386391 PMC5816150

[bib61] Streitner C, Koster T, Simpson CG, Shaw P, Danisman S, Brown JWS, Staiger D (2012) An hnRNP-like RNA-binding protein affects alternative splicing by in vivo interaction with transcripts in Arabidopsis thaliana. Nucleic Acids Res 40: 11240–11255. 10.1093/nar/gks873 23042250 PMC3526319

[bib62] Su YH, Liu YB, Zhang XS (2011) Auxin-cytokinin interaction regulates meristem development. Mol Plant 4: 616–625. 10.1093/mp/ssr007 21357646 PMC3146736

[bib64] Tantikanjana T, Yong JWH, Letham DS, Griffith M, Hussain M, Ljung K, Sandberg G, Sundaresan V (2001) Control of axillary bud initiation and shoot architecture in Arabidopsis through the SUPERSHOOT gene. Genes Dev 15: 1577–1588. 10.1101/gad.887301 11410537 PMC312715

[bib65] Tatapudy S, Aloisio F, Barber D, Nystul T (2017) Cell fate decisions: Emerging roles for metabolic signals and cell morphology. EMBO Rep 18: 2105–2118. 10.15252/embr.201744816 29158350 PMC5709733

[bib66] Vandenbussche F, Petrášek J, Žádníková P, Hoyerová K, Pešek B, Raz V, Swarup R, Bennett M, Zažímalová E, Benková E, (2010) The auxin influx carriers AUX1 and LAX3 are involved in auxin-ethylene interactions during apical hook development in Arabidopsis thaliana seedlings. Development 137: 597–606. 10.1242/dev.040790 20110325

[bib67] Weijers D, Nemhauser J, Yang ZB (2018) Auxin: Small molecule, big impact. J Exp Bot 69: 133–136. 10.1093/jxb/erx463 29309681 PMC5853209

[bib68] Xiao W, Molina D, Wunderling A, Ripper D, Vermeer JEM, Ragni L (2020) Pluripotent pericycle cells trigger different growth outputs by integrating developmental cues into distinct regulatory modules. Curr Biol 30: 4384–4398.e5. 10.1016/j.cub.2020.08.053 32916110

[bib69] Xie MT, Chen HY, Huang L, O’Neil RC, Shokhirev MN, Ecker JR (2018) A B-ARR-mediated cytokinin transcriptional network directs hormone cross-regulation and shoot development. Nat Commun 9: 1604. 10.1038/s41467-018-03921-6 29686312 PMC5913131

[bib70] Xu J, Chua NH (2009) Arabidopsis decapping 5 is required for mRNA decapping, P-body formation, and translational repression during postembryonic development. Plant Cell 21: 3270–3279. 10.1105/tpc.109.070078 19855049 PMC2782270

[bib71] Xu J, Chua NH (2012) Dehydration stress activates Arabidopsis MPK6 to signal DCP1 phosphorylation. EMBO J 31: 1975–1984. 10.1038/emboj.2012.56 22407295 PMC3343339

[bib72] Xu J, Yang JY, Niu QW, Chua NH (2006) Arabidopsis DCP2, DCP1, and VARICOSE form a decapping complex required for postembryonic development. Plant Cell 18: 3386–3398. 10.1105/tpc.106.047605 17158604 PMC1785416

[bib73] Xu C, Luo F, Hochholdinger F (2016) LOB domain proteins: Beyond lateral organ boundaries. Trends Plant Sci 21: 159–167. 10.1016/j.tplants.2015.10.010 26616195

[bib74] Ye L, Wang X, Lyu M, Siligato R, Eswaran G, Vainio L, Blomster T, Zhang J, Mähönen AP (2021) Cytokinins initiate secondary growth in the Arabidopsis root through a set of LBD genes. Curr Biol 31: 3365–3373.e7. 10.1016/j.cub.2021.05.036 34129827 PMC8360765

[bib75] Yokoyama A, Yamashino T, Amano Y, Tajima Y, Imamura A, Sakakibara H, Mizuno T (2006) Type-B ARR transcription factors, ARR10 and ARR12, are implicated in cytokinin-mediated regulation of protoxylem differentiation in roots of Arabidopsis thaliana. Plant Cell Physiol 48: 84–96. 10.1093/pcp/pcl040 17132632

[bib77] Yu X, Li B, Jang GJ, Jiang S, Jiang DH, Jang JC, Wu SH, Shan LB, He P (2019) Orchestration of processing body dynamics and mRNA decay in Arabidopsis immunity. Cell Rep 28: 2194–2205.e6. 10.1016/j.celrep.2019.07.054 31433992 PMC6716526

[bib78] Zhang ZB, Wu YL, Gao MH, Zhang J, Kong Q, Liu YA, Ba HP, Zhou JM, Zhang YL (2012) Disruption of PAMP-induced MAP kinase cascade by a Pseudomonas syringae effector activates plant immunity mediated by the NB-lrr protein SUMM2. Cell Host Microbe 11: 253–263. 10.1016/j.chom.2012.01.015 22423965

[bib79] Zuo Z, Roux ME, Sæmundsson HP, Müller M, Munne Bosch S, Petersen M (2021) The Arabidopsis thaliana mRNA decay factor PAT1 functions in osmotic stress responses and decaps ABA-responsive genes. Febs Lett 595: 253–263. 10.1002/1873-3468.13977 33124072

[bib80] Zuo Z, Roux ME, Rodriguez E, Petersen M (2022a) mRNA decapping factors LSM1 and PAT paralogs are involved in turnip mosaic virus viral infection. Mol Plant Microbe Interact 35: 125–130. 10.1094/mpmi-09-21-0220-sc 35100808

[bib81] Zuo Z, Roux ME, Dagdas Y, Rodriguez E, Petersen M (2022b) PAT mRNA decapping factors function specifically and redundantly during development in Arabidopsis. BioRxiv. 10.1101/2022.07.06.498930 (Preprint posted July 6, 2022).

