# Supplementary material for: The mRNA decapping machinery targets LBD3/ASL9 to mediate apical hook and lateral root development
Source: Life Sci Alliance. 2023 Jun 29;6(9):e202302090. doi: 10.26508/lsa.202302090 (PMC10310928; doi:10.26508/lsa.202302090)
Supplement: Supplementary file 1 [file LSA-2023-02090_TableS1.docx]

**Table S1. Mutants used in this study**

| Mutant | Information | Source |
| --- | --- | --- |
| *path1-4* | 7bp deletion in Exon 2, frame shift and early stop codon | Zuo et al., 2022b |
| *path2-1* | 35bp deletion in Exon 2, frame shift, early stop codon | Zuo et al., 2022b |
| *asl9-1* | SAIL_659_D08, T-DNA insertion in Exon 1 | NASC (Nottingham, UK) |
| *pat1-1* | Salk_040660, T-DNA insertion in Exon 5 | Roux et al., 2015 |
| *summ2-8* | SAIL_1152A06, T-DNA insertion in Exon 1 | Zhang et al., 2012 |
| *dcp5-1* | Salk_008881, T-DNA insertion in 3'-UTR | Xu and Chua, 2009 |
| *dcp2-1* | Salk_000519, T-DNA insertion in Exon 3 | Xu et al., 2006 |
| *arr10-5* | Salk_098604, T-DNA insertion in Exon 5 | Ishida et al., 2008 |
| *arr12-1* | Salk_054752, T-DNA insertion in Exon 3 | Ishida et al., 2008 |
