# Supplementary material for: The mRNA decapping machinery targets LBD3/ASL9 to mediate apical hook and lateral root development
Source: Life Sci Alliance. 2023 Jun 29;6(9):e202302090. doi: 10.26508/lsa.202302090 (PMC10310928; doi:10.26508/lsa.202302090)
Supplement: Supplementary file 2 [file LSA-2023-02090_TableS2.docx]

**Table S2. Primers used in this study**

| **Primer** | **Sequence** | **Use** |
| --- | --- | --- |
| **genotyping** |  |  |
| LBb1.3 | ATTTTGCCGATTTCGGAAC | Genotyping Salk lines |
| SAIL LB3 | CATCTGAATTTCATAACCAATCTC | Genotyping Sail lines |
| SAIL_659_D08 LP | ATGTTGTACGTTGATTTGGGG | SAIL_659_D08 genotyping |
| SAIL_659_D08 RP | TATTCTTTACACGCGGTTTCG | SAIL_659_D08 genotyping |
| DCP2LP | TGATGGGGTTTTGTTTCAGTC | *dcp2* genotyping |
| DCP2RP | ACTATGATCAATGAGTGGCGG | *dcp2* genotyping |
| **qPCR** |  |  |
| ASL9 for | CAAAAGGGTCACAGACACGGAA | qPCR of *ASL9* |
| ASL9 rev | GGCCTCGTACACCATCGAATC | qPCR of *ASL9* |
| EIF4A1F | GATCTGCACCAGAAGGCACA | qPCR of *EIF4A1* |
| EIF4A1R | CCCAGTACCAGACTGAGCCTGTTG | qPCR of *EIF4A1* |
| ARR3F | GAAACTCGCCGACGTGAAAC | qPCR of *ARR3* |
| ARR3R | TCCACAAGCGAAGTTGCAGA | qPCR of *ARR3* |
| ARR4F | ATGGCCAGAGACGGTGGTGTTTC | qPCR of *ARR4* |
| ARR4R | ATCTAATCCGGGACTCCTCATC | qPCR of *ARR4* |
| ARR8F | GACCCAAATGCACTCTCTACATC | qPCR of *ARR8* |
| ARR8R | CTCTTCAGCTCCTTCTTCCAAAC | qPCR of *ARR8* |
| ARR15F | GACGACTGTTGAGAGTGGGAC | qPCR of *ARR15* |
| ARR15R | CTCCTCTGCTCCTTCTATCATAC | qPCR of *ARR15* |
| PIN5-FW | CCATCGGCTCTATTGTCCTTG | qPCR of *PIN5* |
| PIN5-RV | GCGACGAGCACAGGTAGAGA | qPCR of *PIN5* |
| SAUR23 F | ATTCAAACTTTCAGACAAAAGAAATGG | qPCR of *SAUR23* |
| SAUR23 R | ACAAGGAAACAACTCTATCTCTAACT | qPCR of *SAUR23* |
| IAA19 F | GGTGACAACTGCGAATACGTTACCA | qPCR of *IAA19* |
| IAA19 R | CCCGGTAGCATCCGATCTTTTCA | qPCR of *IAA19* |
| TAR2 F | CATGATTTGGCTTACTATTGGCCACAG | qPCR of *TAR2* |
| TAR2 R | GTCTTTCACCAAAGCCCATCCAATC | qPCR of *TAR2* |
| ARR10F | GCTTCTGATGCTGGTTCCTT | qPCR of *ARR10* |
| ARR10R | CAATCACCTTCCGAGAAATCA | qPCR of *ARR10* |
| ARR12F | CTCCACGATGAAGCAGGAA | qPCR of *ARR12* |
| ARR12R | AACTAAACCCTCCATATCCCAAA | qPCR of *ARR12* |
| **5’-RACE** |  |  |
| ASL9 inner | GGCCTCGTACACCATCGAATC | RACE inner PCR |
| ASL9 outer | ATGTCGATGTCACTGTAGAAG | RACE outer PCR |
| EIF4A1 inner | GGTTCTCTTGAAGACCCATGGCATC | RACE inner PCR |
| EIF4A1 outer | CCCAGTACCAGACTGAGCCTGTTG | RACE outer PCR |
